# Changes in body composition of burn patients during the phases of response to trauma

**DOI:** 10.1590/0034-7167-2023-0039

**Published:** 2023-12-04

**Authors:** Andressa Maranhão de Arruda, Patrícia Calado Ferreira Pinheiro Gadelha, Bruna Lúcia de Mendonça Soares, Catiana Coelho Cabral Dowsley, Maria da Conceição Chaves de Lemos, Ana Célia Oliveira dos Santos

**Affiliations:** I Universidade de Pernambuco. Recife, Pernambuco, Brazil; II Hospital da Restauração Governador Paulo Guerra. Recife, Pernambuco, Brazil

**Keywords:** Burns, Nutritional Assessment, Nutritional Status, Body Composition, Bioelectrical Impedance, Queimaduras, Avaliação Nutricional, Estado Nutricional, Composição Corporal, Impedância Bioelétrica, Quemaduras, Evaluación Nutricional, Estado Nutricional, Composición Corporal, Impedancia Bioeléctrica

## Abstract

**Objective:**

To assess the changes in body composition of burn patients through electrical bioimpedance in the phases of response to trauma.

**Methods:**

a longitudinal observational study, carried out from October 2019 to March 2020. Sociodemographic, clinical, epidemiological, anthropometric and body composition data were collected. Statistical analysis was performed with SPSS, considering a significance of 5%. The comparison between variables was performed using the paired Student’s t test.

**Results:**

the sample consisted of 58 adult burn patients, with a mean age of 38.2±12.5 years. The mean body surface area (BSA) with burns was 10.8±7.3%. Nutritional assessment demonstrated a depletion of body weight, Body Mass Index, fat-free mass and muscle mass in the phases of response to trauma (p<0.005).

**Conclusion:**

metabolic alterations in the different phases of the metabolic response to trauma led to a depletion of the nutritional status of burn patients of both sexes during hospitalization.

## RESUMO

**Objetivo:**

avaliar as alterações na composição corporal de pacientes queimados por meio da bioimpedância elétrica nas fases de resposta ao trauma.

**Métodos:**

estudo observacional longitudinal, realizado de outubro de 2019 a março de 2020. Foram coletados dados sociodemográficos, clínicos, epidemiológicos, antropométricos e de composição corporal. A análise estatística foi realizada com o SPSS, considerando significância de 5%. Comparação entre as variáveis foi realizada por meio do teste t de Student pareado.

**Resultados:**

a amostra foi composta por 58 pacientes adultos queimados, com média de 38,2±12,5 anos. A média da área de superfície corporal (ASC) com queimaduras foi de 10,8±7,3%. A avaliação nutricional demonstrou depleção do peso corporal, índice de massa corporal, massa magra e massa muscular nas fases de resposta ao trauma (p<0,005).

**Conclusão:**

alterações metabólicas nas diferentes fases da resposta metabólica ao trauma levaram a depleção do estado nutricional de pacientes queimados de ambos os sexos durante a internação.

## RESUMEN

**Objetivo:**

evaluar alteraciones en la composición corporal de pacientes quemados mediante bioimpedancia eléctrica en las fases de respuesta al trauma.

**Métodos:**

estudio observacional longitudinal, realizado de octubre de 2019 a marzo de 2020. Se recogieron datos sociodemográficos, clínicos, epidemiológicos, antropométricos y de composición corporal. El análisis estadístico se realizó mediante SPSS, considerando una significancia del 5%. La comparación entre variables se realizó mediante la prueba de la t de Student pareada.

**Resultados:**

la muestra estuvo compuesta por 58 pacientes adultos quemados, con una edad media de 38,2±12,5 años. El área de superficie corporal media (ASC) con quemaduras fue de 10,8±7,3%. La evaluación nutricional mostró disminución del peso corporal, índice de masa corporal, masa magra y masa muscular en las fases de respuesta al trauma (p<0,005).

**Conclusión:**

los cambios metabólicos en las diferentes fases de la respuesta metabólica al trauma llevaron a la disminución del estado nutricional de los pacientes quemados de ambos sexos durante la hospitalización.

## INTRODUCTION

Burns are considered a significant public health concern worldwide. The World Health Organization (WHO) has estimated that burn injuries are responsible for approximately 100,800 deaths per year^(1)^. In a study conducted in the United States, it was demonstrated that 75% of burn accidents occurred at home and 13% in the work environment, 95% of which were of an accidental nature^(2)^.

According to the Brazilian Ministry of Health, approximately one million people suffer from burn injuries per year in Brazil^(3)^. The Burn Treatment Center in Brasília reported a major involvement of second-degree burns by flames in males (62%) due to domestic accidents^(4)^.

Among the main consequences of burn injuries is marked hypermetabolism, which results in significant physiological and metabolic changes, particularly muscle and bone catabolism, hepatic steatosis, an increased risk of infections, insulin resistance, multiple organ dysfunction and sepsis. These changes may cause harmful long-term effects^(2,5)^.

Physiological and metabolic disturbances caused by burns cause changes in the body composition of these patients due to the cascade of inflammatory reactions and accentuation of glycolysis, lipolysis and proteolysis^(6)^. Body weight and Body Mass Index (BMI) assessment in critically ill patients and after traumatic events is challenging. The initial fluid replacement in response to the acute event, volume resuscitation during hospitalization, the metabolic cascade, the presence of dressings in the affected areas, and edema are factors that make it difficult to measure, analyze, and interpret the nutritional status and body composition of these patients^(7-9)^.

The use of indirect methods aims at better analysis and interpretation of body changes. Thus, considering the available methods and cost feasibility, we have the use of BIA, a model widely used in clinical practice in hospitalized patients, which offers an easy, quick and practical body compartment and fluid status assessment, enabling the analysis and monitoring of nutritional status and body composition during hospitalization^(10)^.

## OBJECTIVE

To assess the changes in body composition of burns patients through bioelectrical impedance analysis during the phases of response to trauma.

## METHODS

### Ethical aspects

The study was developed in accordance with national and international standards of ethics in research with human beings, in accordance with the Resolution of the Brazilian National Health Council n^o^. 466/12. All study participants were informed about the objectives and benefits of the study and subsequently signed the written Informed Consent Form.

### Study design, period, and place

This is a longitudinal observational study, which adopted the STROBE (EQUATOR) as a framework. It was carried out at a large public hospital specialized in the treatment of burns, from October 2019 to March 2020.

### Population or sample, inclusion and exclusion criteria

The patients included in the study were those who had been assessed within 72 hours after admission, aged over 18 years, from both sexes, with a percentage of ≤50% of the total body surface area (TBSA) affected by burns, who were able to walk in order to undergo anthropometric assessment, and with one of the limbs (upper and lower) from the same side of the body (right or left) unaffected by burns so as to assess body composition by bioelectrical impedance.

Patients with physical limitations that made it impossible to perform anthropometry, bedridden, amputees, pregnant women and carriers of any implantable device, conditions that influence performance and assessment through electrical bioimpedance, were excluded.

### Study protocol

The sample was given for convenience. Sociodemographic, clinical and epidemiological variables of burns were collected by consulting the clinical records available at the service. Sociodemographic data were collected age, sex (male and female), race (white, black and others), origins (urban or rural areas) and education (elementary school, high school and higher education). Data were also collected on the epidemiology: etiological agent (superheated liquids, flammable liquids, electricity, flames, chemicals and others); burn severity (1^st^, 2^nd^ or 3^rd^ degree); nature of injury (accidental, violence and self-harm); place where injured (home, work and outside areas); affected body surface calculated based on the Lund & Browder map; and affected body areas (head, neck, trunk, upper and lower limbs)^(11)^. Clinical data included comorbidities (diabetes mellitus, hypertension and others) and treatment (surgical debridement with and without anesthesia).

Laboratory test, anthropometry and body composition assessment was performed at two moments: within 72 hours of admission and on the 7^th^ day of hospitalization (ebb phase and flow phase of the trauma, respectively).

Information was collected from medical records regarding laboratory tests (glycemia, hemoglobin (Hb), hematocrit (Ht), mean corpuscular volume (MCV), mean corpuscular hemoglobin (MCH), mean corpuscular hemoglobin concentration (MCHC), urea, creatinine, sodium, potassium, albumin, total proteins, C-reactive protein (CRP), total leukocytes and platelets, considering the reference ranges used by the hospital laboratory.

Weight and height were measured according to the original technique recommended by Lohman *et al* ^.(12)^. Participants were weighed using an electric Filizola^®^ digital platform scale, with a maximum capacity of 150 kg and a precision of 100 g, and height was measured using a stadiometer attached to the platform scale with a capacity of 1.90 m and 1 mm accuracy. Weight and height measurements were used to calculate the BMI, and the classification used for adults was that proposed by the World Health Organization (WHO)^(13)^.

Body composition was assessed using tetrapolar bioelectrical impedance analysis (BIA) (Biodynamics model 310^®^). The analysis required at least 4 hours of liquid and/or solid food fasting. During the procedure, a patient lay in a horizontal supine position, without a watch or any other metallic object. The test included the use of 4 electrodes, 2 placed on the dorsum of the hand and 2 on the dorsum of the foot. The variables collected were total body fat percentage (TBF %), basal metabolic rate (BMR; Kcal), fat-free mass (FFM; Kg), fat mass (FM; Kg), total body water percentage (TBW %), skeletal muscle mass (SMM; Kg) and skeletal muscle mass index (SMMI; Kg and %). SMM (Kg) was estimated, for both men and women, from BIA and anthropometric variables, according to the following equation: SMM (Kg) = (height^2^/R x 0.401) + (gender x 3.825) - (age x 0.071) + 5.102, considering height in centimeters (cm), resistance in ohms, sex = 0 for women and sex = 1 for men and age in years^(14)^. SMMI’s estimate and expression in kg and % followed Janssen recommendation, whereby SMMI (%) = (SMM/weight) x 100) and SMMI (Kg/m^2^) = SMM/height^2(15-16)^.

### Analysis of results, and statistics

Statistical analyzes were performed using the Statistical Package for the Social Sciences (SPSS 16.0) (SPSS Inc., Chicago, IL, USA). Data were double-entered and validated. Sociodemographic, epidemiological and clinical variables were presented considering absolute and relative frequencies. Continuous variables were tested for normality of distribution using the Kolmogorov-Smirnov test. Normally distributed variables were described as means and their respective standard deviations, and non-normally distributed variables as median and interquartile ranges (p25 and p75). To analyze the comparison of means, the paired Student’s t test and Wilcoxon t test were used for parametric and non-parametric samples, respectively. A significance level of 5% was used to reject the null hypothesis.

## RESULTS

The sample consisted of 58 adult burn patients, with a mean age of 38.2±12.5 years. According to Table 1, there was a higher frequency of black people (53.4%), from urban areas (65.5%), who had completed elementary school (67.2%), had been affected by superheated liquids (39, 7%), 2^nd^ degree burns (89.7%), of an accidental nature (89.7%) in the home environment (70.7%), regardless of gender. Of the 2^nd^ degree burns observed, 42.3% (n=22) had been caused by superheated liquids and 3^rd^ degree injuries by electricity (n=3; 100%).

**Table 1:** Sociodemographic, clinical and epidemiological characteristics of patients admitted to the Burn Treatment Unit of a referral hospital, Brazil

Variables	n (%)
Sex	
Male	28 (48.3)
Female	30 (51.7)
Race	
White	12 (20.7)
Black	31 (53.4)
Others	15 (25.9)
Originating from	
Urban area	38 (65.5)
Rural area	20 (34.5)
Education	
Elementary school	39 (67.2)
High school	17 (29.3)
Higher education	2 (3.4)
Comorbidities	
Diabetes mellitus	1 (1.7)
Hypertension	9 (15.5)
Etiological agent	
Overheated liquids	23 (39.7)
Flammable liquids	14 (24.1)
Electricity	9 (15.5)
Flames	3 (5.2)
Chemicals	5 (8.6)
Others	4 (6.9)
Severity of injury	
1^st^ degree	1 (1.7)
2^nd^ degree	52 (89.7)
3^rd^ degree	5 (8.6)
Nature of injury	
Accidental	52 (89.7)
Violence	2 (3.4)
Self-harm	4 (6.9)
Where it took place	
Home	41 (70.7)
Work	11 (19.0)
Outside	6 (10.3)
Area of body affected	
Head/neck	22 (37.9)
Trunk	21 (36.2)
Upper limbs	35 (60.3)
Lower limbs	26 (44.8)

Burns caused by flammable liquids resulting from violence were observed in 2 male patients (100%), and self-harm was observed in 3 female patients (100%). The mean TBSA with burns was 10.8±7.3%. In terms of the proposed treatment during the hospitalization period, surgical debridements with anesthesia were performed on 56 patients (96.6%), and without anesthesia, on 36 patients (62.1%).

In Table 2, a significant reduction may be observed in the parameters of Hb (p<0.001), HT (p=0.010), MCHC (p=0.023), urea (p=0.003), creatinine (p=0.021), CRP (p< 0.001) and platelets (p=0.006), from ebb to flow phase.

**Table 2:** Comparison between laboratory tests of burn patients during the phases of metabolic response to trauma, Brazil

Variables	Admission (ebb phase)	7^th^ day (flow phase)	*p* value
Glycemia^**^, mg/dL	88.0 (81.7 - 97.5)	88.0 (82.0 - 92.7)	0.317
HB^*^, g/dL	13.9±1.5	13.2±1.4	<0.001
HT^*^, %	40.6±4.2	39.2±4.3	0.010
MCV^*^, fL	87.9±5.8	88.2±5.4	0.235
MCHC^**^, g/dL	34.1 (33.2 - 35.1)	34.0 (33.0 - 34.6)	0.023
MCH^*^, pg	30.7±2.0	30.7±2.3	0.791
Urea^**^, mg/dL	28.0 (23.0 - 32.0)	29.0 (27.0 - 35.2)	0.003
Creatinine^**^, mg/dL	0.6 (0.5 -0.7)	0.7 (0.6 -0.8)	0.021
Sodium^**^, mg/dL	139.0 (136.0 - 140.0)	137.0 (136.0 - 140.0)	0.284
Potassium^**^, mg/dL	4.2 (3.9 - 4.5)	4.2 (4.0 - 4.5)	0.167
Albumin^**^, mg/dL	3.6 (3.4 - 4.0)	3.5 (3.5 - 3.9)	0.269
Total proteins^**^, mg/dL	6.9 (6.7 - 7.0)	6.9 (6.7 - 7.0)	0.977
CRP^**^, mg/L	6.3 (1.5 - 9.0)	1.2 (1.2 - 5.4)	<0.001
WBC^*^, x10^3^	10.9±3.4	10.1±2.3	0.062
Platelets^*^, x10^3^	267.9±93.6	231.2±65.0	0.006

HB - hemoglobin; HT - hematocrit; MCV - mean corpuscular volume; MCHC - mean corpuscular hemoglobin concentration; MCH - mean corpuscular hemoglobin; CRP - C-reactive protein; WBC - white blood count.

* Paired Student’s t test for normally distributed variables;

** Wilcoxon t test for non-normally distributed variables for variables with non-normal distribution. p<0.05.

Anthropometric measurements and body composition were compared in the ebb and flow phases according to sex (Table 3). A reduction of 1.3 kg was observed in women (p<0.001); 0.5 kg/m^2^ in BMI (p<0.001); 67.3 Kcal in BMR (p<0.001); 0.5% in TBF (p<0.001); 2.3 Kg in FFM (p<0.001); 1.2 kg in SMM (p=0.001); 1.6 kg/m^2^ in SMMI (p=0.003); 0.5% in SMMI (p=0.001); and an increase of 1.7 kg of FM (p=0.017). Males presented a reduction of 1.9 kg (p<0.001); 0.6 kg/m^2^ in BMI (p<0.001); 74.5 Kcal in BMR (p<0.001); 0.8% in TBF (p<0.001); 2.7 kg in FFM (p=0.001); 1.2 kg in MME (p=0.021); 1.7 kg/m^2^ in SMMI (p=0.031); and 0.5% in SMMI (p=0.020). No statistical difference was observed in FM (p=0.362). During the assessed period, there was no change in body water volume distribution.

**Table 3:** Comparison of the Body Mass Index and body composition according to sex during the phases of metabolic response to trauma, Brazil

Variables	Female (n=30)			Male (n=28)		
	Admission (ebb phase)	7^th^ day (flow phase)	*P* value	Admission (ebb phase)	7^th^ day (flow phase)	*P* value
Weight	73.5±15.9	72.2±15.6	<0.001^*^	69.2±11.3	67.3±10.1	<0.001^*^
BMI	28.9±5.4	28.4±5.3	<0.001^*^	24.2±3.8	23.6±3.5	<0.001^*^
BMR	1400.0±224.7	1332.7±218.9	<0.001^*^	1626.9±223.4	1552.4±203.9	<0.001^*^
TBF	37.3±3.1	36.8±6.2	<0.001^*^	18.5±5.9	17.7±6.0	<0.001^*^
FFM	46.2±7.3	43.9±7.0	<0.001^*^	53.8±7.5	51.1±7.1	0.001^*^
FM	26.4±11.1	28.1±10.2	0.017^*^	15.2±7.3	16.0±7.9	0.362
SMM	19.6±3.2	18.4±2.7	0.001^*^	28.2±3.9	27.0±4.0	0.021^*^
SMMI	28.0±5.7	26.4±5.6	0.003^*^	42.4±6.7	40.7±7.6	0.031^*^
SMMI	7.7±1.1	7.2±0.8	0.001^*^	9.9±1.2	9.4±1.2	0.020^*^
TBW	70.8±2.9	70.9±3.0	0.850	71.5±2.5	72.2±3.9	0.166

BMI - body mass index (kg/m^2^); BMR - basal metabolic rate (kcal); TBF - total body fat (%); FFM - free-fat mass (kg); FM - fat mass (kg); SMM - skeletal muscular mass (kg); SMMI - skeletal muscular mass index (kg/m^2^; %); TBW - total body water (%). Paired Student’s t test for normally distributed variables.

* p<0.05.

## DISCUSSION

Victims of burn injuries suffer from clinical, psychological and nutritional repercussions^(17)^. It is known that living conditions based on origin and education are factors that may contribute to a higher risk of accidents. Adult age, elementary education and originating from urban area are factors that may also cause a higher risk for incidents, since these conditions characterize the economically active population and consequently favor greater exposure to risk situations.

A higher frequency of burns on the upper and lower limbs may cause a delay in returning to work, thereby bringing about economic and social problems, in addition to reducing an individual’s functional capacity, enabling the development of a chronic health condition^(18)^.

It is known that the evolution of burn patients involves significant alterations in the immune system and biochemical disorders, which affect morbidity and mortality, mainly prompting an increase in endothelial permeability causing the characteristic edema, protein catabolism, leukocytosis and inflammatory processes^(19)^.

In the present study, it was observed that patients’ laboratory parameters during hospitalization remained stable, probably due to the small extent of burns on BSA (10.8±7.3%), thereby avoiding the occurrence of metabolic alterations capable of reproducing major biochemical repercussions.

There was a notable decrease in Hb, Ht, MCHC and platelets as a consequence of hemolysis after burn injuries, accentuating the systemic response according to the degree and extent of the injury, associated with fluid replacement with subsequent hemodilution and blood loss, when dressings and debridements were performed. The deviation of protein metabolism promoting the formation of a new skin to the detriment of the formation of new red blood cells may also be contributing to a reduction of these parameters. A significant reduction in serum CRP concentration was also observed during hospitalization, reflecting an improvement in the inflammatory profile associated with wound healing and immunological competence.

There are very few studies in the literature that have monitored burn patients’ nutritional evolution due to the presence of fluid disturbances that make it difficult to interpret direct and indirect methods of nutritional assessment. This is the first study conducted in Brazil to assess burn patients during the different phases of the metabolic response to trauma and using BIA. Furthermore, it is also the first study to use the SMM and SMMI estimation formulas recommended by the European consensus on sarcopenia for the population with burns^(20-21)^.

One of the main metabolic characteristics after burns involves increased resting energy expenditure (REE), thermogenesis and loss of muscle mass. Hypermetabolism causes a 3 to 10-fold increase in serum cortisol concentration, which may persist for up to three months after the injury. Furthermore, cortisol inhibits glucose uptake and glycogen synthesis by skeletal muscle, thereby directly and/or indirectly causing muscle mass loss^(4,22-24)^. Biolo *et al*. demonstrated that the vast majority of the body’s protein reserves reside in skeletal muscle, hence, a state of hypercatabolism primarily compromises muscle mass. In fasting burn patients, there is a 1.5-fold increase in the rate of muscle protein synthesis and a 1.8-fold increase in the rate of protein depletion, which encourages a negative nitrogen balance due to a marked loss of skeletal muscle mass^(25)^.

Nutritional status is a relevant factor in patients’ healing and clinical evolution after burns. In the total sample of the present study, the BMI indicated a higher frequency of eutrophy (n=24; 41.9%), followed by overweight (n=21; 36.2%), thus corroborating the study by Silva *et al*.^(26)^. A significant reduction in body weight and BMI was observed in both sexes (p<0.001). However, the BMI parameter is not sufficiently sensitive to indicate precise changes in body compartments, especially in the population with burn injuries due to the presence of fluid disturbances. Thus, regardless of the nutritional diagnosis by BMI, the use of indirect methods should be considered in order to assess changes in the body composition of these patients during hospitalization.

Our data on body composition assessment with the BIA method have indicated a reduction in BMR, FFM, SMM and SMMI in the first phase of the metabolic response to trauma. This phase includes glycolysis, glycogenolysis, gluconeogenesis and proteolysis, increasing protein degradation mainly in muscle tissue for energy supply and tissue synthesis, which thereby explains the depletion of FFM, SMM and BMR in these patients. Furthermore, there is a marked lipolysis, aided by the increase in cortisol and consequent increase in the release of free fatty acids (FFA) for energy supply^(4)^. This condition may even be of even greater concern due to being bedridden during hospitalization, thus inhibiting the synthesis of skeletal muscle mass.

Increased levels of FFA and systemic lipids are commonly associated with a hypermetabolic response. Studies have reported significant lipid alterations and high triglyceride levels in burn injuries with increased negative effects^(27-28)^.

After a marked lipolysis, there is an increase in FFA, which may be used by cells to generate energy or esterify in the liver in the form of triglycerides. However, these esterified triglycerides in the liver are released as low-density lipoproteins (LDL) into the plasma. Therefore, the reasons that explain this increase in triglycerides in burn patients include FFA synthesis stimulation as a product of accentuated lipolysis and by high levels of plasma glucose, increased production of LDL and low plasma levels of high-density lipoproteins (HDL)^(27)^.

In the present study, it was observed that, during their entire hospital stay, patients evolved with an increase in FM. This may be explained by the increase in lipid cycling after burns, regulated by catecholamines, corticosteroids and cytokines, followed by an increase in triglycerides and FFA, suggesting greater decomposition and re-esterification of fats, aided by insufficiency of FFA carriers^(27-29)^. Previous studies have reported central fat accumulation in burn patients due to intense hypermetabolism^(30)^.

FFM is a good conductor of electrical current compared to FM. Thus, patient hydration may influence FFM levels. In turn, SMM better characterizes the gross muscle mass of burn patients, since it is not directly influenced by hydration. It should be noted that TBW did not vary significantly between the assessments. This suggests that increased fluid replacement did not influence the loss of FFM, since its depletion occurred during hospitalization. Studies have reported a linear relationship between height and muscle mass, while body weight has a curvilinear relationship with muscle mass. Thus, height is the best factor for correcting muscle mass in these patients, justifying the significant reduction in SMMI (Kg/m^2^) that occurs during hospitalization^(14-16)^.

The application of recurrent fasting for surgical debridement with anesthesia implies an increase in catabolic hormones, an inflammatory response and a secretion of catecholamines and glucagon, accentuating catabolism and muscle depletion^(31)^. Moreover, there is an increase in triacylglycerol degradation, causing an excess of fatty acids and glycerol in the blood, whereby these substances are consequently stored in the adipose tissue in the form of triglycerides^(27)^. Therefore, in these patients, metabolic and physiological changes during fasting directly contribute to a reduction of food intake, muscle mass depletion and lipogenesis.

### Study limitations

Some limitations should be mentioned, such as the sample size and the formulas used to estimate patients’ SMM and SMMI, which are not specific for the population with burn injuries, thereby suggesting the development and future validity of specific equations for populations that suffer from fluid disturbances.

### Contributions to nursing, health, and public policies

The study can contribute to better targeting the assessment interprofessional analysis of the burned population, seeking to identify early the impairment of the nutritional status of these patients, in order to contribute to plans of interprofessional actions and treatments to improve quality of life, with consequent reduction of health costs.

## CONCLUSION

In conclusion, it was observed that most burn injuries occurred accidentally in the home environment, involving 2^nd^ degree burns, superheated liquids and mainly affecting the upper and lower limbs. The higher frequency of elementary school education and originating from urban areas, thus characterizing the economically active population, reinforces the need for preventive measures based on educational guidelines regarding simple changes in small daily, household habits in order to reduce incidents.

The use of direct and indirect methods of nutritional assessment in patients with severe fluid disturbances may help in providing a better analysis and interpretation of nutritional status evolution. A depletion of body weight, BMI, FFM and muscle mass was observed in the phases of the metabolic response to trauma. This indicates the importance of monitoring changes in the body compartments of these patients so that interprofessional strategies may be adopted early to assist in attenuating metabolic stress and reducing morbidity and mortality.
